# Editorial: Migration, ethnicity, race and diversity in a post-Brexit and pandemic Britain

**DOI:** 10.3389/fsoc.2025.1563314

**Published:** 2025-03-04

**Authors:** Steve Garner, Sin Yi Cheung, Sofia Vougioukalou, April-Louise Pennant

**Affiliations:** ^1^Department of Criminology, Sociology and Social Policy, Swansea University, Swansea, United Kingdom; ^2^School of Social Sciences, Cardiff University, Cardiff, United Kingdom

**Keywords:** race and ethnic inequalities, pandemic, Brexit, historical, structural, institutional inequalities

This Research Topic brings together a diverse range of contributions to examine the challenges and opportunities posed by migration and ethnic/racial inequalities in a post-Brexit and pandemic Great Britain. While the primary focus is on the lived experiences of individuals and communities, the broader historical, structural and institutional injustices are very much at the heart of these new investigations.

Two articles in this Research Topic are large-scale survey-based pieces on the pandemic; one on Welsh Twitter's engagement with the Brexit referendum; and the other an analysis of the economic impact of the pandemic on ethnic minorities in the Manchester area. A further article examined the relationship between social capital and mental wellbeing. Also included in this Research Topic is an opinion piece on theorizing the race state; and a theory article on the concerted action of social movements to impede anti-racist campaigns and scholarship in France and the United States, whose focus on the struggle over the terms of debate is particularly relevant to Great Britain since 2016.

The review by Wiśniowski et al. focused on the economic impact on ethnic minority communities in the Greater Manchester area, highlighting differences before, during and after COVID 19. In a similar vein, Li and Ding examined the impact of different types of social capital, namely, networks of contact, support and trust, on the wellbeing of minority ethnic groups from different social classes in the Great Britain.

Interestingly, Li and Ding found that all three of the domains of social capital they identified improved well-being scores when considered in isolation. However, when they were all included at once, the outcomes worsened. In other words—too much effort and sacrifice in COVID times lead to poorer outcomes. The article concludes that there were no specific ethnic impacts. In fact, Li and Ding's summary is deceptively concise: “the root cause of disadvantage thus lies in socio-economic inequality rather than ethnicity, but ethnicity is a notable bearer of such inequality.”

What is the relationship between socioeconomic inequality and ethnic inequality in this era? The answer is that there is more than one relationship. Anyone who has worked on ethnic or racial disadvantage will find the articles in this Research Topic to be an enlightening read. Wiśniowski et al.'s fascinating compilation of data covers inter alia, universal credit take-up, food, financial and housing security, encouraging us to reflect on the ultimate conclusion about ethnicity “bearing” socio-economic inequality. For a long time, patterns of wealth, poverty and advantage/disadvantage in the Great Britain have demonstrated an “ethnic penalty” (Cheng and Heath, 1993) for ethnic minorities, and that this penalty has particular ethnicised consequences for the distribution of opportunity, wealth and poverty (Garner and Bhattacharyya, 2011; Piccitto et al., 2024). Even within ethnic groups there are patterns of wealth and poverty, and this is the norm from decades of study. So, when the conclusion is phrased as “socio-economic inequality rather than ethnicity” (Li and Ding) it suggests a binary or causal relationship, which is, we would argue, an over-simplification.

As social scientists, binaries are not usually our friends. They are not a random pattern of socio-economic outcomes. How people got into the various deciles when we started data collection is not a given, so the statement that socioeconomic status is more important than ethnicity relies on starting the clock at the beginning of the data collection rather than understanding that patterns of discrimination and relative advantage have produced these departure points, and shaped the evolution of the outcomes analyzed in this piece. Indeed, Wiśniowski et al. (p. 12) argue that:

“…ethnic minorities have faced worse labor market outcomes due to the pandemic and restrictions than White people across the UK. The government's provisions have not included enough consideration for prior inequities in the labor market and the ways in which ethnic minority groups were uniquely impacted by the pandemic because of these.”

These “prior inequities” are key to decoding the pandemic experience: the disproportionate number of ethnic minority workers in frontline, precarious and essential services, obliged to use public transport and at higher risk of living in poorer housing exposed them to a higher likelihood of contracting COVID and also to worse labor market impacts at the end of the pandemic.

This entanglement of the socioeconomic with race and ethnicity leads us to Ogunrotifa's opinion piece critiquing a high-profile US theoretical intervention on race: Emirbayer and Desmond (2015). This book has received much criticism from race scholars, particularly for its minimal engagement with the perspectives of scholars of color, and for writing a prescriptive tract on the putative paucity of theory in a field in which neither has expertise. Ogunrotifa's critique identifies a lack of connection to the workings of capitalism, i.e. a failure to theorize in context. He points out that the process of racialisation is not mentioned in the book, and notes that contemporary forms of racism are more varied in their foci than just the body (as it is assumed by Emirbayer and Desmond). This could also be a critique of basing definitions of racism on ideology alone. While no critical race scholar can work without acknowledging the material and economic dimensions of the actors and the relationships that they study, Ogunrotifa's final framing, like that of the network of scholars he cites, claims the theoretical primacy of the material (economic) over the cultural (race). Nothing can be resolved by attention to racial democracy and multiculturalism he argues, because capitalism will continue to strain these social organizing principles in times of crisis.

This is obviously a challenging, although far from new proposition for those of us invested in less deterministic frameworks, with race at their center. Indeed, this tension between class and race—both as conceptual tools and as sources of experience—has existed since the early days of the sociology of racism, and has brought us landmark pieces of scholarship such as Cox's Caste, Class and Race (Cox, 1948), Robinson's Black Marxism (Robinson, 1983), Miles' Racism and Migrant Labor (Miles, 1982) and ultimately the “racial capitalism” paradigm (Bhattacharyya, 2018). Ogunrotifa's contribution to this Research Topic is a part of this tradition.

Communication and shared ideas link the other two articles in this Research Topic. The first attempted to identify patterns in Welsh Twitter users' engagement with the EU Referendum. It was refreshing to see a Welsh focus on Brexit research, and indeed political engagement with immigration, so this was a step in a good direction. With all the caveats about representativeness, Peixoto Gomes sought to discover whether Welsh and English Twitter demonstrated similar or distinct patterns of discourse on Brexit. Her study reveals a good deal of complexity. While both focused on politics and nation more than anything else, the Welsh emphasis was less on immigration and when it was, it was more positive than the English. However, the connections between race, immigration, the economy and support for the two voting options did not appear to be particularly strong for either, in this sample.

Finally, Garner's contribution goes beyond the Great Britain to link two ongoing campaigns led by the state and supported by social movements, aiming to push back against anti-racist advances, as slim as they may be, in the United States and France. Opposition to anti-racist scholarship and all things associated with it have a long history in the United States, and opposition to Muslim immigration in Metropolitan France could similarly be dated back generations. However, the central state in France and State governments in the United States have never before engaged so proactively and critically with the concepts and the scholars themselves as they have in recent years. Patterns of discourse shape the French state's critique of the concept of Islamophobia and the American states' critique of what they shorthand as “Critical Race Theory.” Such attacks are also funded, organized and engaged in by non-state actors, and this moment is worth identifying in the long arc of the struggle against racism, as an important watershed. The United Kingdom has witnessed a similarly fraught engagement with the official definition of Islamophobia, and institutional racism in recent years. The work in this Research Topic enables us to touch on a number of issues that run through contemporary analyses of race and ethnicity: the material inequalities embedded in British society that the pandemic has exacerbated; the fine distinctions within the Great Britain over Brexit (which of course remain to be studied further); the longstanding argument over how to ultimately resolve social inequalities that sits behind all race scholarship; and the state's newfound populist urge and ability to engage in ideological warfare with the proponents of antiracist paradigms, portraying them as battles over the soul of the nation.
